# Spatial mapping of low pressure cluster jets using Rayleigh scattering

**DOI:** 10.1038/s41598-023-32373-2

**Published:** 2023-04-18

**Authors:** Milaan Patel, B. R. Geethika, Jinto Thomas, Hem Joshi

**Affiliations:** 1 Institute for Plasma Research, Near Bhat, Gandhinagar, Gujarat 382428 India; 2 Homi Bhabha National Institute, Training School Complex, Anushaktinagar, Mumbai, 400094 India

**Keywords:** Fluid dynamics, Optical techniques, Macromolecules and clusters

## Abstract

In this work, we report evolution of atomic clusters in a highly under-expanded supersonic jet of Argon. A high resolution and sensitive Rayleigh scattering based experimental set-up is designed to overcome the limitations encountered in conventional set-ups. Further, the measurement range could be extended from a few nozzle diameters to 50 nozzle diameters. Simultaneously, we had been able to generate 2D profiles of the distribution of clusters inside the jet. This paves the way to track the growth of clusters along the flow direction experimentally, which until now was limited to few nozzle diameters. The results show that spatial distribution of clusters inside the supersonic core deviates considerably from the prediction of the free expansion model. We exploit this to estimate cluster growth along the expansion direction. Further, it is observed that the growth of the clusters gets saturated after a certain distance from the nozzle. At the jet boundary, we see substantial cluster strengthening immediately upstream of barrel shock while the normal shock exhibits disintegration of clusters. These observations are noticed for the first time, which, we believe will further the understanding of cluster dynamics in a supersonic jet.

## Introduction

Cluster formation in supersonic free expansion is important in generating isolated molecules for laser plasma experiments^1^, generation of fast electrons and ions^2^, conversion of high-energy charge particles into neutral atoms^3^ and generating superhot microplasma for laser fusion experiments^4^. Furthermore, the information regarding cluster formation is an important aspect in the study of photo induced chemical reactions^5^. Clusters are formed when free expanding jet undergoes isentropic cooling along the flow and the temperature of the gas reaches below the condensation point. During this, the gas molecules get coagulated due to weak nuclear force and form clusters. Relatively large (typically $10^2$–$10^5$ atoms) with atomic density equivalent to liquid/solid of cluster is advantageous for better coupling of laser energy with neutrals in laser cluster experiments^1,4^. These experiments depend critically on the size of the cluster which can be controlled by operating conditions of the jet (mainly reservoir temperature and density). Despite a number of works on the clusters in the last two decades^6–9^, accurate prediction of their size still remains a grey area. Further, the cluster size reported from different experiments differs by at least an order of magnitude under similar experimental conditions^8,9^. The well-known Hagena parameter^10^ and its modified version^11^ are simple empirical relations used to predict the cluster size for a particular nozzle operating at particular reservoir density and temperature. However, its predictions often differ from the experimental measurements^12^ which indicates further scope of including more parameters which are responsible for cluster growth. It is well known that the size of the cluster ($N_c$) increases with the reservoir pressure ($P_0$) according to power law $N_c \propto P_0^{\beta }$, where for Argon $\beta =2.0{-}2.5$^13–15^. However, recently it has been reported that the cluster may also grow along the flow axis^16^. This axial growth can be the reason behind the errors in different experiments as the axial distance for size measurements is typically chosen randomly based on the convenience. Further, cluster size estimated by model of Hagena does not take into account the distance from the nozzle. Hence, it becomes important to study cluster growth dynamics along the expansion.

The adopted techniques to measure cluster size can be broadly categorized as scattering^6,9,11^, interferometry^12^, infrared absorption imaging^17^ and ionization. Ionization method is most robust, however it is invasive by nature. Hence it has inherent measurement inaccuracies. Moreover, it is more suitable for molecular beams rather than for jets^18^. Infrared absorption imaging can only be applied to certain gases which have significant absorption in a particular range. Interferometry, although is non-intrusive, can only be employed close to the nozzle exit because it is less sensitive for rarefied flows at longer axial distances. As a result, scattering-based approaches are generally preferred. The choice of scattering signal (Mie, Rayleigh, or Raman) depends on the system’s sensitivity as well as on the size of the clusters. Thus, each has its own advantages and limitations. For instance, Mie scattering requires wavelength of incident light to be smaller than the size of the cluster^8^ and Raman scattering cannot be applied to monoatomic gas. Rayleigh scattering is hence ideal for low pressure cluster jets. The accuracy in measuring the cluster size using Rayleigh scattering depends on the calibration. In many reported works, calibration is carried out by assuming the cluster size at the onset of the signal to be 100 atoms^12^. This approach is not universal as the detector sensitivity and light collection geometry vary for different setups. This vague assumption during the calibration can result in inaccuracy in the estimation of the cluster size^11^. To circumvent this, Reyleigh scattering technique is often combined with interferometry^11,12^. However, as mentioned earlier interferometry can only be used for measurements close to the nozzle. This limits the effective measurement range of the combined setup to a few nozzle diameters. Hence, Rayleigh scattering approach used to map cluster density profile needs to be independent as well as sensitive enough to measure low-density at relatively longer distances from the nozzle. Interferometric Rayleigh scattering technique has also been successfully demonstrated for turbulence velocity measurements^19^.

As the evolution and dynamics of cluster growth at longer distances is very important, we significantly improved the sensitivity of Rayleigh scattering system. We adopted a sonic nozzle and the total density is estimated using free expansion model which does not have the need of supplementary approach to measure total density, thus making Rayleigh measurements independent. In our experiments, we use a pulsed ns Nd-YAG laser with high gain ICCD camera to image the spatial distribution of the scattering signal from the cluster. We improved our density detection limit to $10^{20}$ molecules/cu.m by reducing the background significantly. This enabled us calibrate the detectors using neutral gas atoms in the pressure range same as inside the jet. This significantly improves the accuracy in the measurement of the size of clusters. Further with increased sensitivity, we are able to detect the clusters up to a distance of 50 times the nozzle diameter. To the best of our knowledge, the spatial mapping for such a long spatial extent from the nozzle has been performed for the first time in this work. This gives better insight into the cluster dynamics in the jet core as well as its boundaries. The measurements are carried out for argon jets due to its relatively higher Rayleigh scattering cross section and higher possibility of cluster formation.

In the subsequent sections the experimental setup is described in section “Experiment setup”, measurement method is described in section “Measurement method”, mathematical approach is given in section “Theoretical approach”, results are discussed in section “Results and discussion” and section “Conclusion” concludes the work.

## Experiment setup

**Figure 1:**
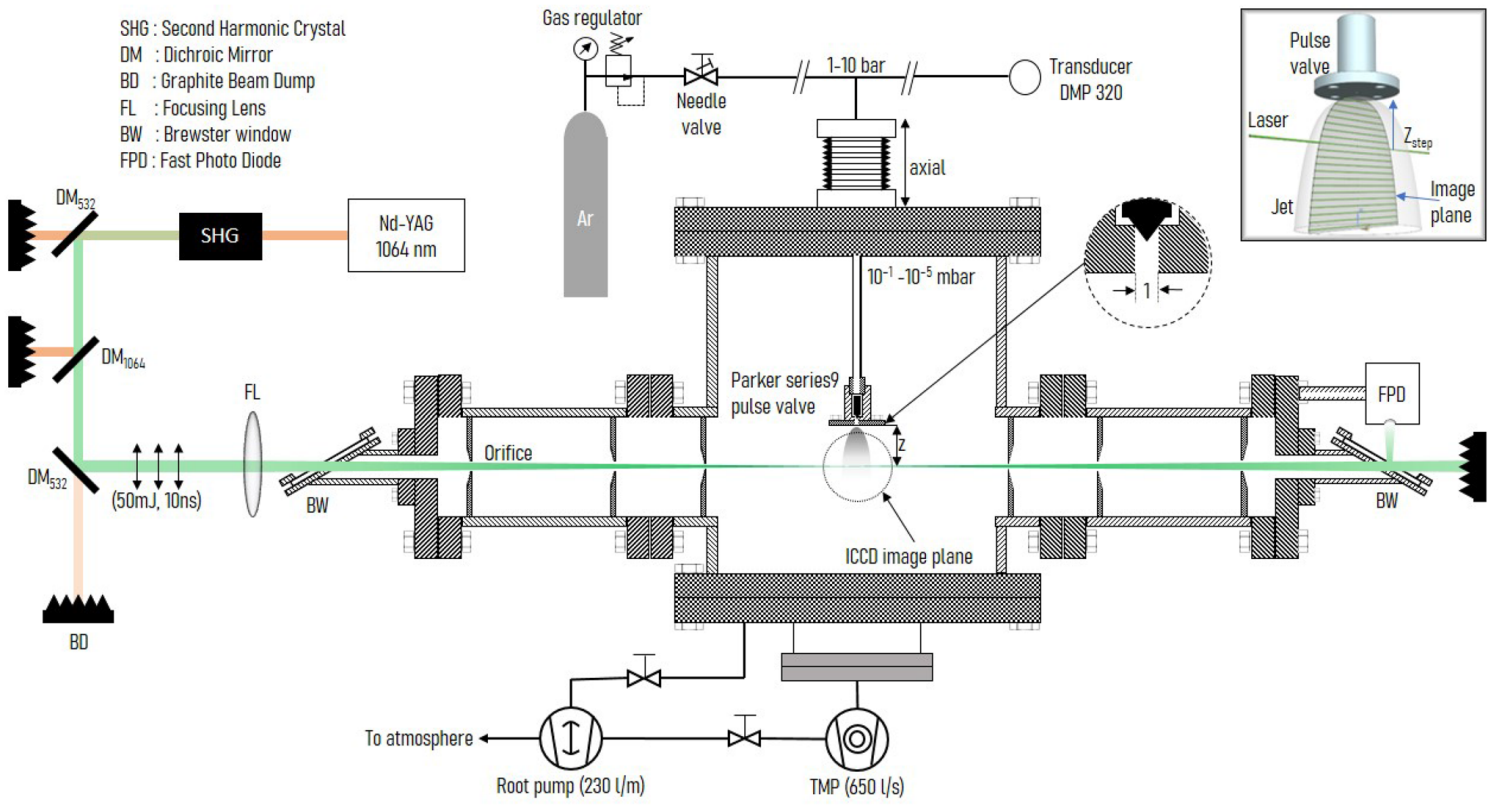
Schematic view of the experimental setup used to generate spatial profile of clusters in the argon jet. Top-right inset shows the scanning technique used to generate 2D profiles of the cluster jet.

The experimental setup for jet imaging is shown in Fig. 1. It consists of a supersonic jet source inside the the vacuum vessel. Jet axis is oriented vertically and the laser is aligned horizontally perpendicular to the jet axis. Illuminated laser path due to the scattering from jet is imaged using intensified CCD camera whose optical axis is aligned perpendicular to the jet axis. The experimental system can be sub-divided into vacuum vessel and pumping system, gas feed system, laser and alignment optics and imaging optics. These subsystems are explained in details in subsequent discussion.

Vacuum vessel is pumped by a Pfieffer Hi-pace700 turbo-molecular pump (TMP, 650 *l*/*s*) mounted at the bottom (DN 160 CF flange) of the vessel and a Pfieffer ACP-15 dual stage root pump (230 *l*/*m*) is used as backing pump to the TMP. Root pump is also used for initial rough vacuum generation ($\simeq \, 0.05$ mbar) inside the vessel. Pressure inside the vacuum vessel is monitored by Pfieffer PKR251 combined full range gauge (for pressures below $10^{-2}$ mbar, accuracy 30%) and MKS instruments micro-pirani gauge calibrated for argon (for pressure above $10^{-2}$ mbar, accuracy 10%).

A Parker series 9 pulse valve with 1 mm orifice is used as the source of gas feed. The orifice acts as a sonic nozzle. It is mounted inside the vessel using 1/4 inch SS-tube via UHV gas feedthrough. External end of the feedthrough is connected to a high pressure gas supply line. The feedthrough is mounted on a bellow type manually controlled UHV translation stage for axial positioning of the valve with respect to laser path. Ultra high purity (99.99%) argon gas is supplied to the valve at pressure ranging from 1–6 bar through high pressure gas supply line. Pressure inside the gas supply line is regulated by a precision needle valve and is monitored using a diaphragm type pressure transducer (DMP 320) having 1% accuracy.

A pulsed nanosecond Nd-YAG laser (Continuum Powerlite DLS 9030, 1064 nm) having 10 ns pulse for 1064 nm at 30 Hz is used as the laser source. Pulsed laser is preferred over continuous laser due to certain advantages. First, the laser pulse duration is small, hence the integration time of imaging system can be kept as short as laser pulse width ($\simeq 10$ ns) without sacrificing the signal. This provides measurement which is nearly instantaneous in flow time scale. Secondly, compared to a continuous laser, a pulsed laser can achieve high intensity in short bursts giving intense scattering signal. In the experiments, we use second harmonic (532 nm) of the Nd-YAG laser to further enhance the intensity of scattered light (Rayleigh scattering intensity ($I_{RS}$) depends inversely on fourth power of wavelength ($I_{RS} \propto 1/\lambda ^4$). Moreover, second harmonic of Nd-YAG is in the visible region of the spectrum which also helps in the alignment of laser. A set of di-chroic mirrors for 1064 nm and 532 nm is used to filter out the fundamental of Nd-YAG. Energy of second harmonic (532 nm) beam is set to 50 mJ per pulse. Any measurement error in cluster size estimation comes from the measurements of scattered photons (counts) which is due to the fluctuations in laser energy and camera noise (dark current) both of which are inherent properties of the laser and camera respectively. Fluctuation of laser energy among individual pulses is monitored using a fast photo diode (FPD). We ensured that the variation is always less than 5% by maintaining the ambient temperature of the laser, second harmonic crystal and associated optics within $\pm \, 1^{\circ }$C. By averaging over 100 shots, the standard deviation is further reduced below 1%. The dark current of the detector is relatively lower compared to the signal amplitude essentially having rather small contribution except at the far expansion region (> 45 mm) where the signal strength is low. Moreover, measured parameters are time averaged values over 10 ns which is small relative to the flow time. Hence, statistical fluctuations are likely to from the flow itself. The cumulative effect of these is below 10% which can be considered as the accuracy of the measurement.

Laser is focused inside the vacuum vessel directly below the valve using a plano-convex lens of 2 m focal length placed outside the vacuum vessel. The spot size of the focused laser beam is $\simeq \, 0.5$ mm which determines the spatial resolution. As the focal length is very larger compared to the lateral width of jet (few cm), The scattering volume is nearly cylindrical with diameter equal to spot size. Scattered light is collected by an ICCD camera (Princeton Instruments PI max 2) with inbuilt pulse time gate (PTG) for fast imaging and micro-channel plate (MCP) for higher gain (up to $10^4$). Each pixel in the camera subtends a square of side $\simeq 135\, \upmu$m on the image plane. With this arrangement, laser makes 4 pixel wide image on the camera. Hence, it is the laser spot size, instead of camera, that ultimately determines the spatial resolution.

Sensitivity of the detection system is an important parameter which has to be improved for detecting small clusters in low pressure jets. Use of short integration time for a pulsed laser system suppresses background due to ambient light but background due to the scattering of laser beam itself is the major factor which limits the sensitivity of the detection system. This scattering can arise from the diffusive light from the dust particles (suspended due to dynamic environment in the chamber during the gas injection), diffusion of light from the glass window, as well as back reflection from the surfaces of the optics used to align and focus the laser beam. We tried to minimize the laser background with precise alignment, by using Brewster windows at the entrance and the exit of the laser to the vacuum vessel. Positioning these windows very far (1.5 m) from the region of interest and shielding the diffused light from the window with multiple orifices placed along the beam also helps remove scattered background light. With this, we are able to reduce the background sufficiently to record the scattering signal from neutral argon atoms for number density as low as $10^{20}$ N./cu.m. We have calibrated ICCD using neutral argon gas to determine the geometry constant ‘*k*’ (Eq. (5)) of the detection system. As can be seen from the results, the cluster size is at least an order of magnitude smaller than the wavelength of the laser. Hence, we are always within the range of Rayleigh scattering regime. Additionally, collisional broadening which results from Brillouin scattering affects the profile (Intensity vs wavelength) of Rayleigh scattered photons. This can result in the enhancement of the Rayleigh signal due to additional contribution from Brillouin photons. However, the contribution of Brillouin scattering only comes when the mean free path of the gas is comparable or smaller than the wavelength^20,21^. For $\lambda =532$ nm, Brillouin contribution may affect if the number density is higher than $2\times 10^{24}$ No./cu.m. The total density is always smaller than the limiting value in our measurements. Hence, contribution of Brillouin scattering can be safely ruled out. Doppler broadening effects which are dominant in high pressure turbulent jets^19^ are also insignificant in present case due to low expansion temperature. It is important to note that, for a cluster jet, Rayleigh signal due to the scattering from cluster is always greater than that in case of than atoms. Hence, contributions of scattering associated with atoms and their broadening effects are insignificant.

## Measurement method

To decrease the background filling, the jet is operated in short bursts by opening the pulse valve for 1.5 ms at 10 Hz using IOTA-One pulse valve controller. The background pressure increases initially due to dynamic gas injection and stabilizes after a few seconds based on the average gas injection rate established by the nozzle pressure and pumping rate of TMP. The pulse length is adjusted from 1 to 5 ms depending on the nozzle pressure ($P_0$) to produce various working background pressures ($P_b$) at a steady pumping rate inside the vacuum vessel. Due to dynamic injection, small dynamic pressure fluctuation (5%) is observed. This variation, however, is less than that of the Pfieffer ($\pm \, 30\%$) and MKS gauges ($\pm \, 10\%$) absolute measurement accuracies. Furthermore, fluctuations disappear when $P_b$ exceeds 1 mbar and the background pressure remains stable under dynamic injection. Since the orifice of the pulse valve itself acts as a sonic nozzle, for stable background condition, jet boundaries grow and decay at the start and the end of the gas injection cycle. Thus, within individual gas injection cycle there is a period of stable flow. Time required for mechanical opening of the pulse valve and to establish steady flow in the Parker series 9 pulse valve is smaller than 1 ms^22^. Hence, measurements are recorded in steady flow condition by synchronizing the laser within 1–1.5 ms region of the input gas pulse.

**Figure 2:**
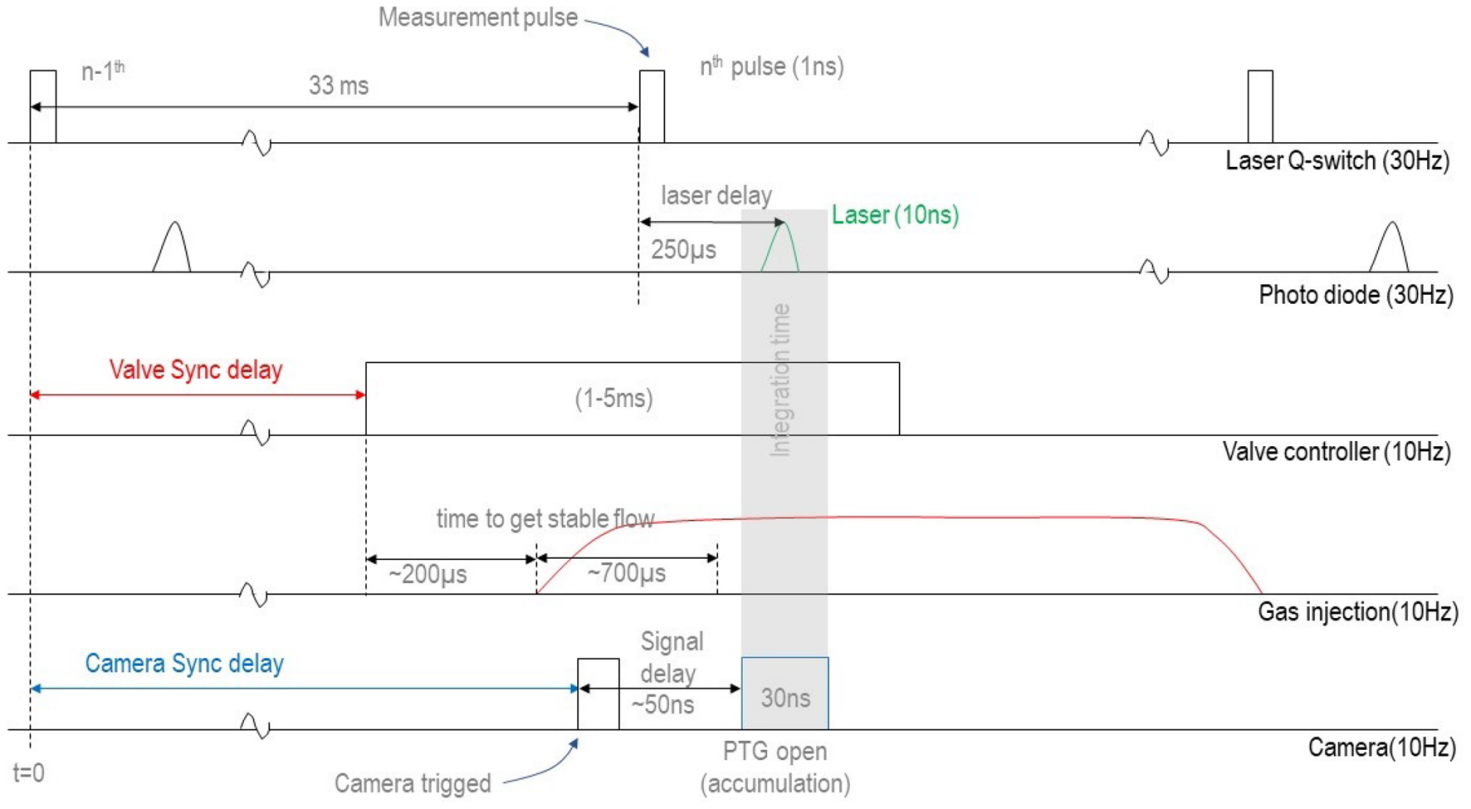
Timing diagram showing synchronization of laser and camera with gas injection.

Figure 2 shows the timing diagram for synchronizing the laser and camera with the jet. Pulse valve controller is triggered from the trigger out of the laser. Time delay between the trigger out from the laser and laser pulse is $250\,\upmu$s. However, time to establish steady flow in the pulse valve is higher than $250\,\upmu$s. As a result, pulse valve is required to trigger prior to the laser pulse itself. This is achieved by operating the pulse valve at a lower frequency (10 Hz) than that of the laser (30 Hz). Hence, for every pulse of the jet there are two additional pulses of laser where the pulse valve closed. Hence, trigger to the pulse valve can be taken from the laser pulse 33 ms prior to the one that is to be synced with the jet. Trigger delay is adjusted to synchronize the laser with the gas injection such that measurement is done when steady flow of jet is achieved (1 ms).

ICCD camera is triggered at 10 Hz using the trigger from the pulse valve controller and a preset delay is added to the trigger to synchronize the opening of PTG with the laser pulse. The opening duration of the PTG which is also the integration time of ICCD is set to 30 ns to envelop the 10 ns laser pulse. Each frame of the video captured by the camera operating at 10 Hz is the image of the laser light scattered from the jet at a particular axial location from the nozzle. At that axial location, the intensity (count) of the scattered signal is averaged over 100 frames to minimize the effects of the fluctuation in laser energy. Measurements are done at different spatial locations along the jet axis by moving the UHV translation stage of the valve in steps of 1 mm (top left inset of Fig. 1). Measurements close to the nozzle (< 4 mm) are not possible as the reflections from the pulse valve body saturates the high gain detectors. The gain of the ICCD camera is set from the nozzle pressure to get signal at maximum possible distance from the jet without saturating the ICCD for measurements near the nozzle. Nevertheless, a single axial scan of the jet profile is always carried out at fixed gain of the ICCD. Same approach is used to record and subtract the background by performing the measurements without gas injection. Captured images of the jet at a particular distance give the radial profile of the jet at that distance. The images captured at different axial distances are stitched together to generate the profile of the cluster jet.

## Theoretical approach

Rayleigh scattering intensity for an incident light of intensity $I_0$ from $n \cdot V$ molecules in volume *V* and solid angle $\Omega$ is given by Eq. (1)^11^.

1 $$\begin{aligned} I_{RS}\propto I_0 \frac{d\sigma }{d\Omega }\Omega n V \end{aligned}$$

where *n* is the number density of the scattering species (gas or cluster), *V* is the scattering volume and $d\sigma /d\Omega$ is the differential scattering cross-section. For plane polarized light of a particular wavelength, the differential scattering cross-section at $90^{\circ }$ in a plane perpendicular to the plane of polarization depends on the size of scattering molecule (Eq. 2)^12^.

2 $$\begin{aligned} \left| \frac{d\sigma }{d\Omega }\right| _{90^{\circ }} \propto r^6 \end{aligned}$$

Considering the cluster as sphere, the radius (*r*) of the cluster can be related to number of atoms per cluster ($N_c$) as $r^3 \propto N_c$. Using Eqs. (1) and (2), Rayleigh scattering intensity in terms of $N_c$ and cluster density ($n_c$) is given by Eq. (3).

3 $$\begin{aligned} \left| I_{RS}\right| _{90^{\circ }} \propto I_0 \Omega V n_c N_c^2 \end{aligned}$$

The duration of the laser pulse is fixed (10 ns) in all the experiments and also the laser beam size. Thus, for a single pulse, we can safely replace the intensity (*I* counts s$^{-1}$ m$^{-2}$) with total counts ($C_{RS}$). Scattering volume and solid angle remain same for each individual pixel of ICCD and laser energy is maintained within 5% during calibration and experiments. Hence, $I_0$, *V* and $\Omega$ can be combined with proportionality constant and can be taken as geometry constant ‘*k*’. Furthermore, we omit angle $90^{\circ }$ for the sake of clarity. With these modifications, Eq. (3) can be simplified to Eq. (4). This is commonly used relation to estimate cluster size from scattered signal^12,13^.

4 $$\begin{aligned} C_{RS} = k n_c N_c^2 \end{aligned}$$

The value of geometric constant ‘*k*’ can be estimated by measuring the scattering from the neutral atoms at different background pressures of the vessel by considering them as clusters of 1 atom size. Thus during calibration number density of cluster ($n_c$) is replaced with neutral density ($n_a$) (which is known from the background pressure) and $N_c$ becomes $N_a$ in this case, which is taken as 1. The calibration condition is shown in Eq. (5). It simplifies to well-known liner relationship of scattered photons vs neutral gas density, typically observed in a Rayleigh scattering signal. It should be noted that non-linear effects due to extremely low temperatures will not be present for the jet because the total temperature of the jet (static + dynamic) stays the same as that of the reservoir.

5 $$\begin{aligned} C_{RS\,(neutral)} = k n_a \end{aligned}$$

Equation (4) assumes that all the atoms in the jet have formed clusters. This cannot be realized in underexpanded jets in general as only fraction of molecules forms clusters. The fraction of molecules forming clusters can be represented as the liquid-mass fraction (*g*)^11^ which is the ratio of atoms forming clusters to the total number of atoms. Mathematically, *g* is given by $\frac{n_c \cdot N_c}{n(z,\theta )}$, where $n(z,\theta )$ represents the total density (cluster + neutral) at measurement point located at a distance z from throat with angular position $\theta$ w.r.t nozzle axis. Hence for cluster jets, Rayleigh scattering signal is sum of the signals from the cluster and neutrals as shown in Eq. (6).

6 $$\begin{aligned} C_{RS} = k ~g N_c ~n(z,\theta ) ~+~ k ~(1-g) N_a ~n(z,\theta ). \end{aligned}$$

In the subsequent discussion regarding calibration curve we see that contribution from neutrals is not significant relative to the cluster. Hence, Rayleigh scattering for a cluster jet is given by Eq. (7).

7 $$\begin{aligned} C_{RS} = k ~g N_c ~ n(z,\theta ). \end{aligned}$$

In this work, $C_{RS}$ is determined from the spatial profile of Rayleigh scattering measured experimentally, *k* is determined from calibration and $n(z,\theta )$ is estimated from the free expansion model of Biejerinck^23^ as shown in the Eq. (8). where, $\dot{N}$ is the flow rate, $u_\infty$ is the terminal velocity, $\gamma$ is the ratio of specific heats at constant pressure and volume, $\kappa$ is the “peaking factor” (1.98 for monoatomic gas), *b* is the angular factor (3 for monoatomic gas) and $\theta _{PM}$ is the angle of Prandtl–Meyer expansion fan for sonic nozzle. To account for the effects of sudden contraction and boundary layer formation in the finite length of the orifice, flow rate is measured experimentally using approach described in Appendix-2^24^. For pulse duration of 1–5 ms, $\dot{N}$ is 45% of the theoretical value (calculated using 1D isentropic approach^23^) and the orifice diameter which results in experimental value of flow rate is 0.6 mm. The absolute density determined by free expansion model with this approach has been substantiated experimentally^24^.

8 $$\begin{aligned} n(z,\theta )&= \frac{\kappa \dot{N}}{u_{\infty }\pi z^2} cos^b\left( \frac{\pi }{2}\frac{\theta }{\theta _{PM}}\right) \nonumber \\ \theta _{PM}&= \frac{\pi }{2} \left[ \left( \frac{\gamma +1}{\gamma -1}\right) ^{1/2}-1\right] \end{aligned}$$

Equation (7) thus gives the value of $g \cdot N_c$ at each spatial location of the jet. This value represents the combined effect of the cluster size and its fraction among the neutrals. In other words, it represents the deviation in $C_{RS}$ from the free expansion trend $n(z,\theta )$. Note that, at any location, instantaneous values of *g* and $N_c$ can increase due to the formation of new cluster by coagulation/coalescence or decrease due to the fragmentation of clusters through collisions. The net effect, however, would be such that the product $g \cdot N_c$ will increase along the expansion direction as cooling of jet favors more cluster formation. As a result, deviation in $C_{RS}$ relative to $n(z,\theta )$ would be towards the increasing trend of either/both liquid mass fraction or/and cluster size as long as the flow is collisional.

It is rather difficult to predict the value of *g* and $N_c$ independently at different spatial locations. This has often lead to many researchers to assume $g=1$ to estimate $N_c$^9,12^. However, this would be a vague assumption for low pressure room temperature jet in our case^11^. Considering these discrepancies, we believe the product $g \cdot N_c$ would be scientifically accurate representation of our results.

## Results and discussion

This section is divided into subsections based on the experiment steps. In the first step we estimate the geometric constant using the calibration. In the second, constants of power law are estimated by measuring the scattering intensity at different reservoir (nozzle) pressures at different axial distances from the nozzle. Finally, in the third and last step spatial distribution of $g \cdot N_c$ is calculated using Eq. (7). Additionally, we measure the cluster profiles at different background pressures at a constant nozzle pressure to see its behavior at the jet boundary.

### Calibration

**Figure 3:**
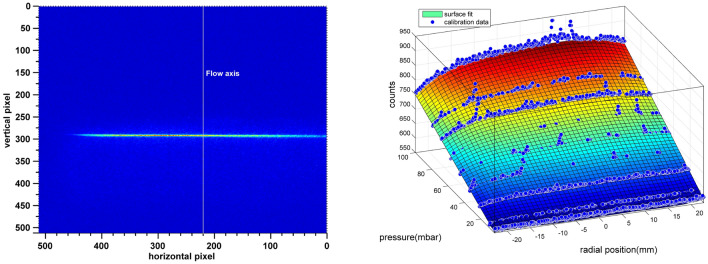
Left panel shows Rayleigh scattered image of the laser beam at background pressure of 100 mbar. Right panel shows the Pressure-intensity plots at different radial position. Geometric constant k is determined for different radial position by fitting a polynomial surface which is linear along the pressure axis and quadratic along the radial axis.

Calibration is done by measuring the scattered intensity at different background pressures in the vessel ranging from 1 to 100 mbar. Figure 3 (left) shows the raw image of the scattered laser light from argon at $P_b =100$ mbar. Out of the focus causes the intensity of laser to fade towards the edges. The flow axis is shown by a vertical white line (with a minor offset to avoid damaged vertical pixels at x = 280 line). To avoid adverse effects of scattering from the valve body during calibration, the pulse valve is shifted far away from the laser (it is not visible in the image). Intensity of the scattered light is averaged over the width of the laser (4 pixels) to generate intensity vs pressure plots displayed in Fig. 3 (right) at different pixel positions. The horizontal pixel values are scaled to the absolute values of the length in the radial direction. Value of geometric constant (*k*) for each radial position is calculated by fitting a linear-quadratic surface to the data points. This compensates the difference in the scattered intensity produced by the change in differential scattering cross-section due to the tilt in optical axis of the individual pixel relative to the optical axis of all pixels of the camera combined. To minimize round off error during curve fitting, slope is determined in terms of counts/mbar and then translated to counts/no.density by multiplying a conversion factor $K_B$ (Boltzmann constant) $\times$ $T_{cal}$ (calibration temperature). It should be noted that decrease in the slope close to the edge is due to inherent misalignment between the spherical focal plane of the camera and linear path of the laser (spherical abrasion). This misalignment deteriorates further when we move camera closer (because of increase in the curvature of the focal plane) which results in the loss of the radial information. As a result, the camera had to be situated farther (0.5 m) from the laser to prevent loss in radial information. A better sensitive camera is consequently necessary to compensate for the loss in signal due to the decrease in solid angle caused by moving the camera away. Thus, there appears to be a trade-off between the total measurable radial span and the detection sensitivity.

### Power law

**Figure 4:**
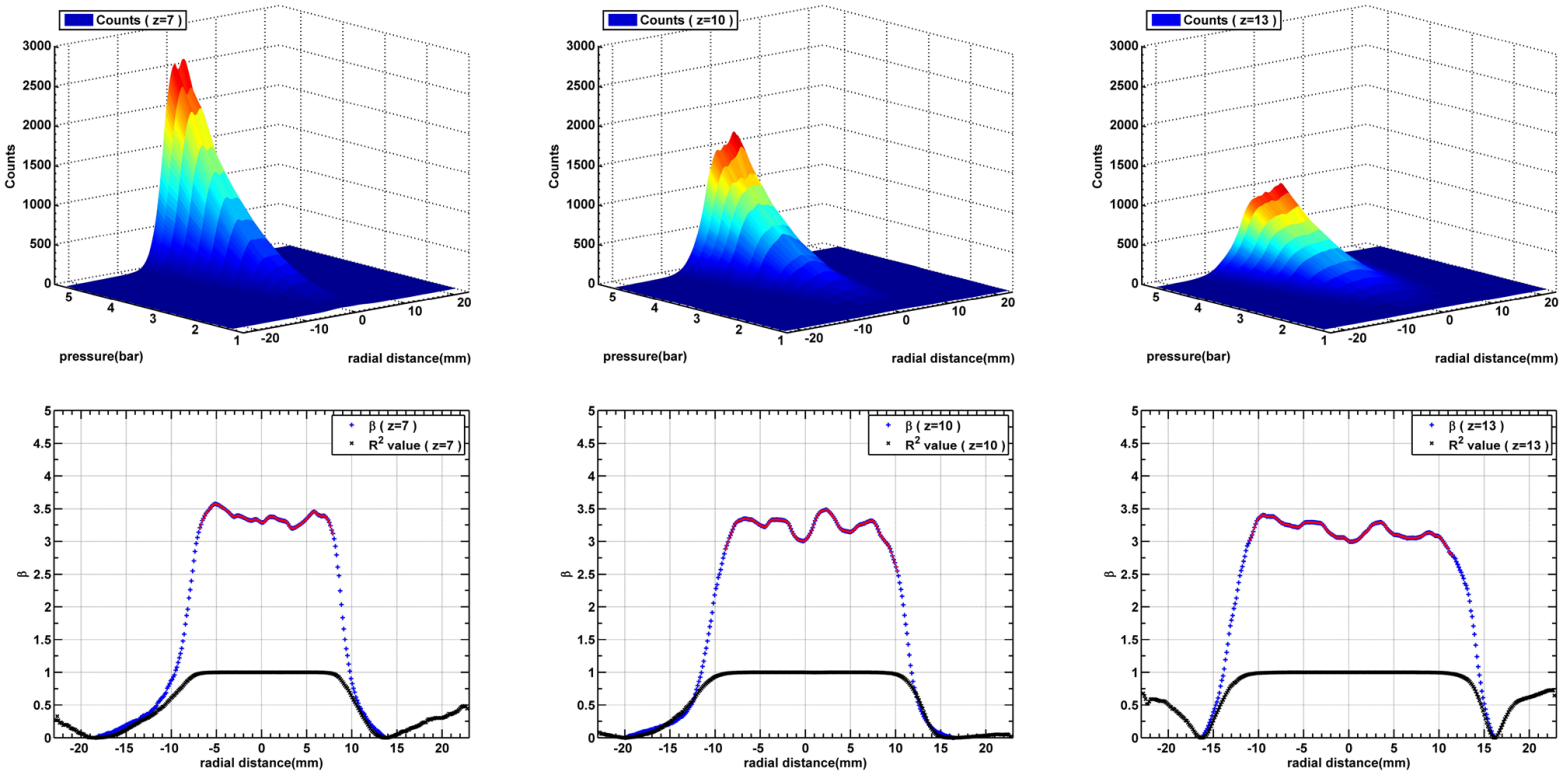
Pressure vs intensity curves at three different distances from the nozzle. It can been seen that at all the three distances co-efficient of power law fit along the radial direction is approximately $3.25 \pm 0.25$. Here, only those values of $\beta$ (masked in red) whose goodness of power law fit (denoted by $R^2$) is greater than 0.95 are considered.

The most appropriate way to ensure cluster formation is by measuring the scattered signal from the jet at different nozzle pressures. In the absence of cluster formation, increase in the nozzle pressure will linearly increase Rayleigh signal as absolute value of total number density at any location inside the jet is linearly dependent on the reservoir pressure. However, when clusters are formed, it has been reported that scattering signal follows power law $C \propto P_0^{\beta }$, where $\beta =3.0{-}4.0$ for argon jets^13–15^.

Figure 4 shows nozzle pressure vs Rayleigh scattering intensity for three different axial locations viz. 7, 10 and 13 mm from the nozzle. Measurements at each axial location include complete radial widths of the jet. As expected, along the axial direction, the radial spread of the jet increases and the scattering intensity decreases as the total density decreases due to expansion. Power law is fitted for each radial location and the corresponding values of $\beta$ are shown in the graphs below the pressure intensity plots (lower panels). We also see that $R^2$ values give confidence in the power law fit. Inaccuracies near the edges are expected as the signal falls to the background level and only those values of $\beta$ for which $R^2>0.95$ can be considered accurate. These are masked in red dots. It is interesting to see that, for the most part of the jet along the radial direction (where the signal to background ratio is at least 10), and for all the three axial locations, the values of $\beta$ is $3.5 \pm 0.2$ are in line with the values reported for Argon by different researchers measuring at different locations along the axis. It is to be noted that the value of $\beta$ is consistent axially as well as radially. This indicates that cluster growth with nozzle pressure is consistent for all the locations within the jet.

### Spatial profile of the clusters

**Figure 5:**
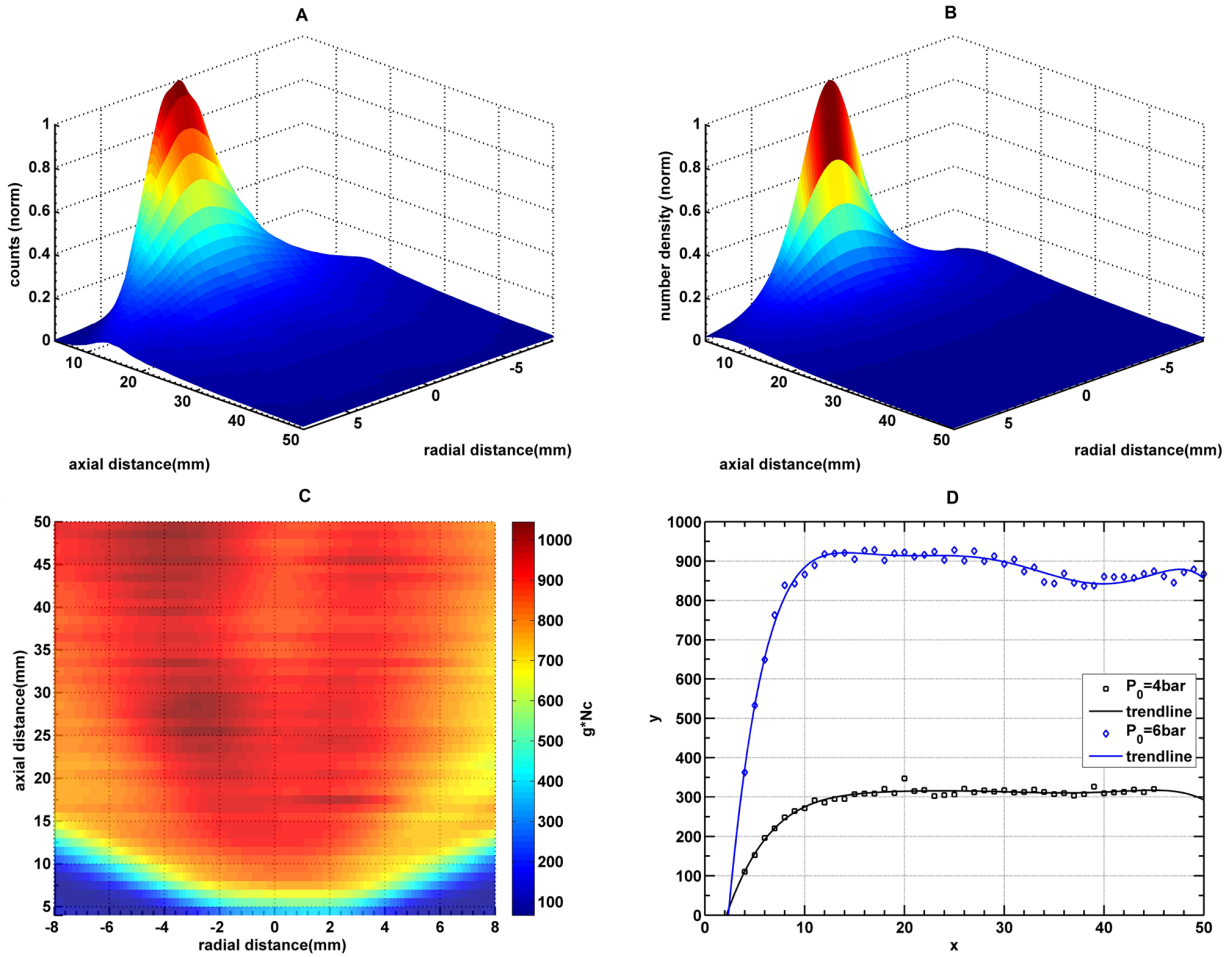
Graph (**A**) shows the spatial distribution normalized Rayleigh signal $C_{RS}$, graph (**B**) shows normalized values of total number density $n(z,\theta )$, graph (**C**) absolute value of the product of liquid mass fraction and cluster size ($g \cdot N_c$) for nozzle pressure $P_0= 6$ bar and graph (**D**) shows the values of $g \cdot N_c$ on the axis for two different nozzle pressure. It can be seen that cluster formation continues along the expansion until it saturates near $z = 10$ mm from the nozzle (Drop in values of $g \cdot N_c$ for $P_0:6$ bar at 35 mm is an experimental artifact).

Figure 5 shows the normalized spatial profiles of scattering counts (A) and total number density (B) calculated using Eq. (8) for $P_0=6$ bar. It can be seen that $n(z,\theta )$ drops with distance as per inverse square law, while $C_{RS}$ does not follow it. This indicates that the factor $g \cdot N_c$ increases along the expansion direction. Figure 5C shows the spatial profile of the $g \cdot N_c$ and Fig. 5D shows the axial trends plotted for two different pressures 6 bar and 4 bar. These two figures apparently represent the most important results of the present work. The trend suggests that more neutral atoms are coagulating to form clusters, either due to the growth of the existing clusters or by the formation of new clusters. This effect seems to be prominent adjacent to the nozzle because of higher density and collision frequency. Surprisingly, growth/formation saturates after 15 mm from the nozzle, which might be due to decreased collisional frequency. The distance for saturation is the same for jets with two different nozzle pressures. However initial growth rate is faster in the jet for higher nozzle pressure, and the absolute values of $g \cdot N c$ also saturate at much higher values due to more coagulation favored by higher neutral density.

The Hagena parameters estimated by scaling law of Hagena^10^ for nozzle pressures of 4 bar and 6 bar come out to be 4205 and 5047 respectively. Using Hagena parameter, one can estimate the theoretical value of liquid mass fraction for from Tao’s work^11^ which is approximately 0.15 for both the cases. It should be noted that $g=0.15$ is derived from the Hagena parameter, which does not account the axial growth. As a result, the scaling law provides a constant value of *g* regardless of axial location. Our results, on the other hand, show that *g* can vary along the axis. Apparently, Hagena model corresponds to data from a single axial location near the nozzle exit. As a result, it only makes sense to compare our data to the predictions of Hagena model in the area near the nozzle exit. The values of $g \cdot N c$ in the vicinity of nozzle exit for $P_0=4$ bar is 105 which results in the cluster size of 700*atoms*/*cluster* for $g=0.15$. Similarly, cluster size for $P_0=6$ bar at the nozzle exit is 2400 atoms/cluster. Estimated cluster sizes calculated using scaling law^25–27^ are 965 atoms/cluster and 1481 atoms/cluster for $P_0 = 4$ bar and 6 bar respectively. This shows that the size of the clusters near the nozzle exit is in fairly good agreement with the estimation from scaling laws. However, improvement in the scaling laws is required to account for the growth along the axis. Furthermore, it is difficult to conclude at this juncture that the liquid mass fraction should stay constant. Hence its variation must also be accounted for accurate description of the cluster formation. As it is not possible to measure the liquid mass fraction independently in our experiment and proposing a modified model is beyond the scope of current work, the size data are presented in the form of $g \cdot N c$.

### Clusters at the jet boundary

**Figure 6:**
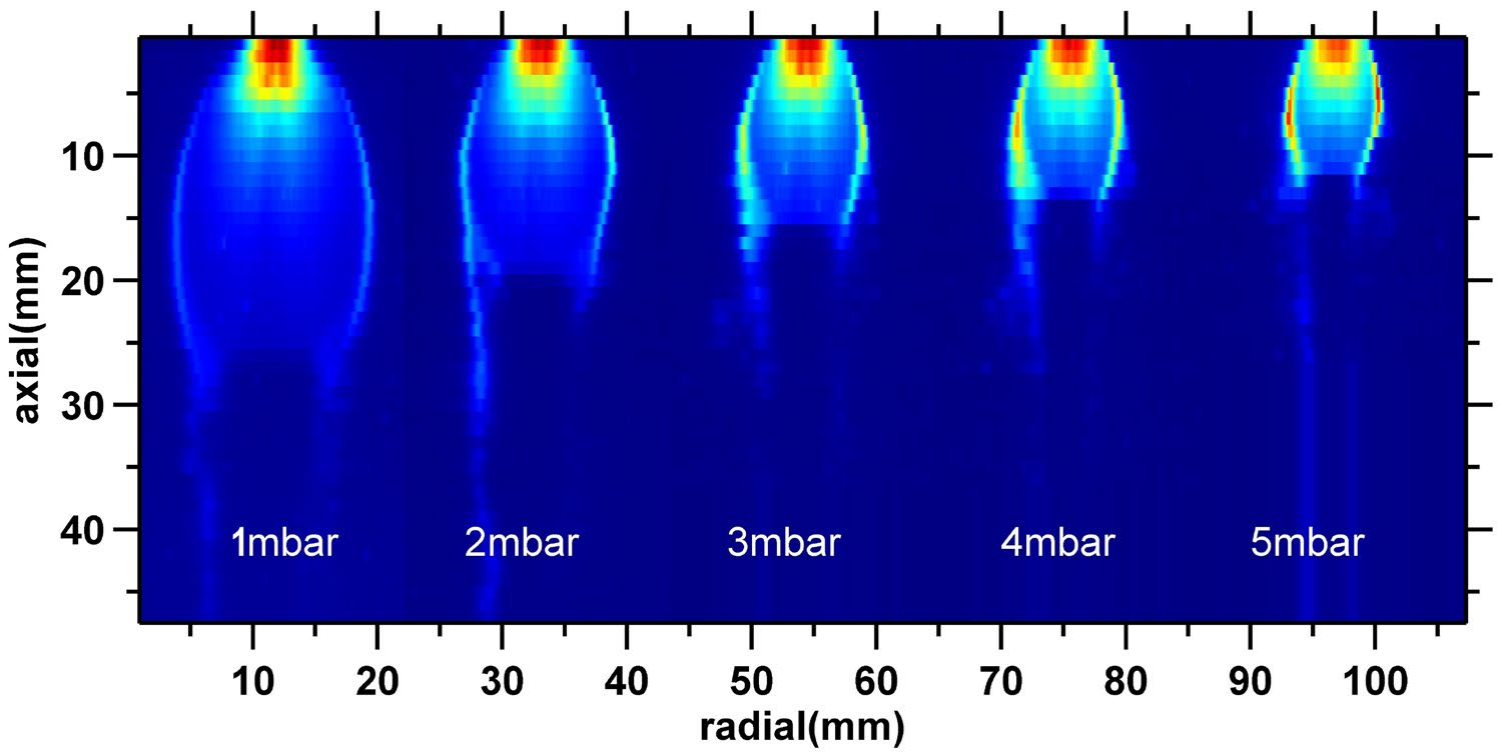
Scattering from the clusters present in the jet operating at five different values of background pressures and same nozzle pressure (4 bar). Individual images of jet are generated by stitching the horizontal stripes of Rayleigh scattering intensity at different distances from the nozzle. Each strip is an average of 100 frames generated from 100 individual jet pulses. The axial resolution of the data is 1 mm (given by scanning step of manual translation stage) and the radial resolution of data is 130 $\upmu$m from the pixel size of ICCD. The colorbar in the image are scaled to absolute values of $C_{RS}$. The image of jet is 0.5 mm thick slice of the jet on the axial plane.

Figure 6 shows spatial mapping of the cluster jet carried out at constant nozzle pressure of 4 bar for different background pressures ($P_b$) ranging from 1 to 5 mbar. $P_b$ values are maintained relatively higher than in the size measurement experiments, because, for background pressures lower than 1 mbar, jet boundary becomes diffused due to smaller collision frequency and could not be observed. Color represents the absolute values of Rayleigh scattering counts. The scattering signal seen in the images is only due to the contribution of clusters. The image clearly shows that the core of the jet is surrounded by the jet boundary which appears as a region of large scattering from the dense cluster. The location where the normal shock is expected, appears as region of low intensity. This is primarily because the temperature and collision frequency in the normal shock disintegrates the fragile clusters. Interestingly, the location of the normal shock matches closely with the predictions of empirical model of Ashkenas and Sherman $z/d_{r}=0.67\sqrt{P_0/P_b}$^28^, and Young’s theoretical approach $z/d_{r}=C\,(\gamma )\sqrt{P_0/P_b},~C\,(\gamma )=0.72~for~argon$^29^ for nozzle diameter of 0.6 mm estimated from the flow rate. It is interesting to see that the emission intensity is higher at barrel shocks, while it is completely absent at normal shocks. This indicates that clusters are present in significant number at barrel shocks. We believe this effect may be related to absolute temperature of the gas at shock regions. At normal shock, flow becomes subsonic which increases the static temperature of the gas. For argon jet, temperature at shock front ($Mach = 1$) is 224 K which is greater than its condensation temperature 87.3 K. As a result, clusters cannot exist at normal shock, thus they appear as low intensity regions in the image. However, at oblique shock region the jet slows down progressively along the barrel shock and jet plume (region between barrel shock and background stream)^30^. In the jet plume, even though Mach number is less than that in the core, flow remains supersonic and becomes subsonic only after jet plume. It is possible that Mach number in the plume is such that the temperature remains lower than the condensation temperature of the argon. We do not have an exact estimate of Mach number inside the transition region but for flow temperature to be lower than condensation limit (87.3 K), Mach number should be greater than 2.71 (calculated from 1D isotropic equation for $T=87.3$ K). This is a possible number for a highly under expanded jet. This enables the clusters to survive in the transition region. Since the density in the transition region is greater than that in the core, scattering signal is stronger.

These images of cluster jet also show the profile of highly under-expanded low pressure jets. Since, it is extremely difficult to generate the profiles of highly under-expanded jets through sub millimeter size sonic nozzle due to small size and rarefied nature of the expansion, visualization techniques are also limited. Typically flow visualization is carried out by capturing emission from the atoms from the atoms excited by electron impact ionization^30–32^. Since the emission volume is the entire volume of the jet, Abel-inversion is required to generate accurate 2D profiles of the jet. Nonetheless, present work gives the flow visualization using Rayleigh scattering technique. Moreover, the imaging volume is the axial plane with the same thickness as the laser stream ($0.5\, d_{nozzle}$). Hence, data do not require post processing to get 2D profiles. The present work also demonstrates a different approach to flow visualization.

## Conclusion

In this paper we report an improved and standalone Rayleigh scattering experiment system to generate the spatial profile of the cluster jet of Argon at long axial and radial distances from the sonic nozzle. Calibration of the imaging system is done using neutral atoms at background pressure same as inside the jet to improve the accuracy. The total density values (cluster + neutrals) required to estimate cluster size are estimated using established free expansion model for the flow rate measured experimentally. Enhancement in cluster formation is reported in terms of the product of liquid mass fraction (*g*) and the size of cluster ($N_c$) to remove the inaccuracies that may arise due to rather vague assumptions of *g*. The spatial profile of cluster size ($g \cdot N_c$) is reported up to the length of 50 times the nozzle diameter. We observe that enhancement in cluster along the expansion direction continues upto a certain distance as long as the density is sufficient after which it eventually saturates. This enhancement may justify the cause of disagreement in the size predictions of clusters from different experiments reported earlier. However, amount of data available on the cluster size of argon, which are required for a reasonable comparison are limited to only a few nozzle diameters as compared to our data which are at substantial distance beyond the nozzle exit. Hence direct comparison of cluster size could not be made. The profile of the clusters at jet boundaries indicates complete disintegration of cluster at the normal shock due to shock temperature higher than condensation limit of argon. On the contrary, clusters exist at barrel shock resulting in increased density, which could be due to flow temperature remaining lower than the condensation limit in the region between inner weak shock and outer strong shock (mixing layer). At present, we cannot comment critically on this observation and believe more finer work is needed to have a comprehensive discussion. While precise details of these tendencies deserve additional investigations, we believe present work will advance our understanding of the mechanism of the cluster formation. The observation of these cluster jet patterns has been possible due to major improvement in the sensitivity and resolution of the experimental system. In brief, in the present study we report Rayleigh scattering for estimating cluster size in argon supersonic jet. For the first time we are able to map cluster density up to a fairly longer distance which has its importance in various applications.

## Data Availability

The data that support the observations of this study are available from the corresponding author upon reasonable request.
